# Predicting microbial interactions with approaches based on flux balance analysis: an evaluation

**DOI:** 10.1186/s12859-024-05651-7

**Published:** 2024-01-23

**Authors:** Clémence Joseph, Haris Zafeiropoulos, Kristel Bernaerts, Karoline Faust

**Affiliations:** 1 Department of Microbiology, Immunology and Transplantation, Rega Institute for Medical Research, Laboratory of Molecular Bacteriology, KU Leuven, 3000 Leuven, Belgium; 2 Department of Chemical Engineering, Chemical and Biochemical Reactor Engineering and Safety (CREaS), KU Leuven, 3001 Leuven, Belgium

**Keywords:** Flux balance analysis, Metabolic modelling, Microbial interactions

## Abstract

**Background:**

Given a genome-scale metabolic model (GEM) of a microorganism and criteria for optimization, flux balance analysis (FBA) predicts the optimal growth rate and its corresponding flux distribution for a specific medium. FBA has been extended to microbial consortia and thus can be used to predict interactions by comparing in-silico growth rates for co- and monocultures. Although FBA-based methods for microbial interaction prediction are becoming popular, a systematic evaluation of their accuracy has not yet been performed.

**Results:**

Here, we evaluate the accuracy of FBA-based predictions of human and mouse gut bacterial interactions using growth data from the literature. For this, we collected 26 GEMs from the semi-curated AGORA database as well as four previously published curated GEMs. We tested the accuracy of three tools (COMETS, Microbiome Modeling Toolbox and MICOM) by comparing growth rates predicted in mono- and co-culture to growth rates extracted from the literature and also investigated the impact of different tool settings and media. We found that except for curated GEMs, predicted growth rates and their ratios (i.e. interaction strengths) do not correlate with growth rates and interaction strengths obtained from in vitro data.

**Conclusions:**

Prediction of growth rates with FBA using semi-curated GEMs is currently not sufficiently accurate to predict interaction strengths reliably.

**Supplementary Information:**

The online version contains supplementary material available at 10.1186/s12859-024-05651-7.

## Background

Microorganisms interact with one another, thereby forming complex interaction networks. Knowledge of these networks is necessary to understand community dynamics and steer it toward a desired behavior. However, it is a challenge to infer microbial interaction networks from abundance data [1], and only a few such networks have been fully resolved to date experimentally (e.g. [2]). In recent years, metabolic modelling has emerged as a new technique to address this problem [3–5].

In metabolic modeling, the knowledge about a cell’s biochemistry is represented in a concise format known as a genome-scale metabolic model (GEM). A GEM incorporates a matrix that stores the stoichiometric coefficients of the substrates and products of all the known biochemical reactions of an organism. GEMs can be constructed automatically from annotated genomes [6–8] or built in a work-intensive process of manual curation [9]. The former approach is much faster but results in GEMs of lower quality. Low-quality GEMs can contain dead-end metabolites (i.e., metabolites that are neither substrates of internal reactions nor excreted), gaps (missing reactions), missing enzyme-reaction links, mass or charge imbalances or futile cycles (irreversible reactions coupled in a cycle). MEMOTE is a recent tool that checks GEM quality systematically [10].

Once a GEM has been obtained, it can be analyzed to learn more about the cell's metabolic capabilities. Flux balance analysis (FBA) is a constraint-based optimization method and a popular technique to computes fluxes through the biochemical reactions assuming that intracellular metabolites are at a steady state, i.e., the production rate of each metabolite equals its consumption rate [11]. FBA maximizes a certain objective function and returns the flux of each reaction (flux vector) when the flux of the objective function equals its optimal. However, while the optimal value of the objective function is unique, the corresponding flux vector may have an infinite number of possibilities [12]. Usually, the objective function maximizes the flux through the (artificial) biomass reaction, which represents cell maintenance and growth [13]. While maximizing for the objective function, the number of non-zero fluxes can be minimized (parsimonious FBA, abbreviated as pFBA[14]). In this case, the objective value is the same as in the standard FBA but the number of required enzymes and, thus, the cost of enzyme production are minimized. Further constraints on flux values can be specified to implement thermodynamic constraints, such as irreversibility of reactions, or to represent limited metabolite concentrations in the medium. In FBA, the medium is defined constraints on fluxes through import reactions (uptake rates).

Given the stoichiometric matrix represented by the GEM, FBA predicts a flux distribution and the growth rate of an organism, where the latter is the flux through the biomass reaction. This is useful in several applications, such as medium optimization, the design of knock-out mutants producing target metabolites, and the exploration of metabolic responses to altered conditions [15]. However, because of its steady-state assumption, FBA is static by default and thus cannot model batch processes. This restriction is circumvented in dynamic FBA, which combines FBA with the differential equations of a kinetic model [16]. The kinetic model describes the changes in biomass and metabolite concentrations, which are updated with growth, consumption, and production rates computed with FBA, in turn providing new uptake rates for the next FBA iteration.

Recently, several tools have been developed that rely on static or dynamic FBA variants to model microbial communities. The definition of a community objective function is still an open problem [17]. As discussed previously [18], most of the tools developed so far can be divided into three groups depending on their solution to this problem, namely (1) introduction of a group-level objective function to optimize the community growth rate [19–21], (2) optimization of the growth rate of each species independently of the others [22–25], and (3) reliance on measured abundances to adjust species growth rates [3, 19].

Reverse ecology aims to learn as much as possible about the ecology of an organism or group of organisms from their genomes [26]. Community FBA is a powerful reverse ecology approach capable of predicting ecological interactions between species pairs from their genomes. This is achieved by building a GEM for each species and then computing growth rates alone and in the presence of another species in silico. Following Gause's famous strategy [27], the comparison of growth rates in mono- and in co-culture then elucidates the interaction sign and strength. For instance, two species that compete will both grow better in mono- than in co-culture, whereas a species cross-feeding on metabolites produced by another will grow better in co- than in monoculture. FBA-based interaction prediction was previously applied to study interactions as a driver of microbial co-occurrence [28], investigate the prevalence of interaction types [4] and their resilience to nutrient change and invasion [5], as well as the synthesis of novel metabolites in the presence of interaction partners [29]. Despite this range of applications, a systematic evaluation of its accuracy has not yet been performed to date. Thus, our goal here is to assess the accuracy of interaction prediction by community FBA tools.

To evaluate interaction prediction accuracy, we collected interactions measured in vitro in 6 studies investigating human and mouse gut microbial communities [2, 30–34]. GEMs were obtained either from AGORA, which is a repository of semi-refined metabolic reconstructions for gut bacteria [35] or from the literature for high-quality reconstructions [36–39].

**Fig. 1:**
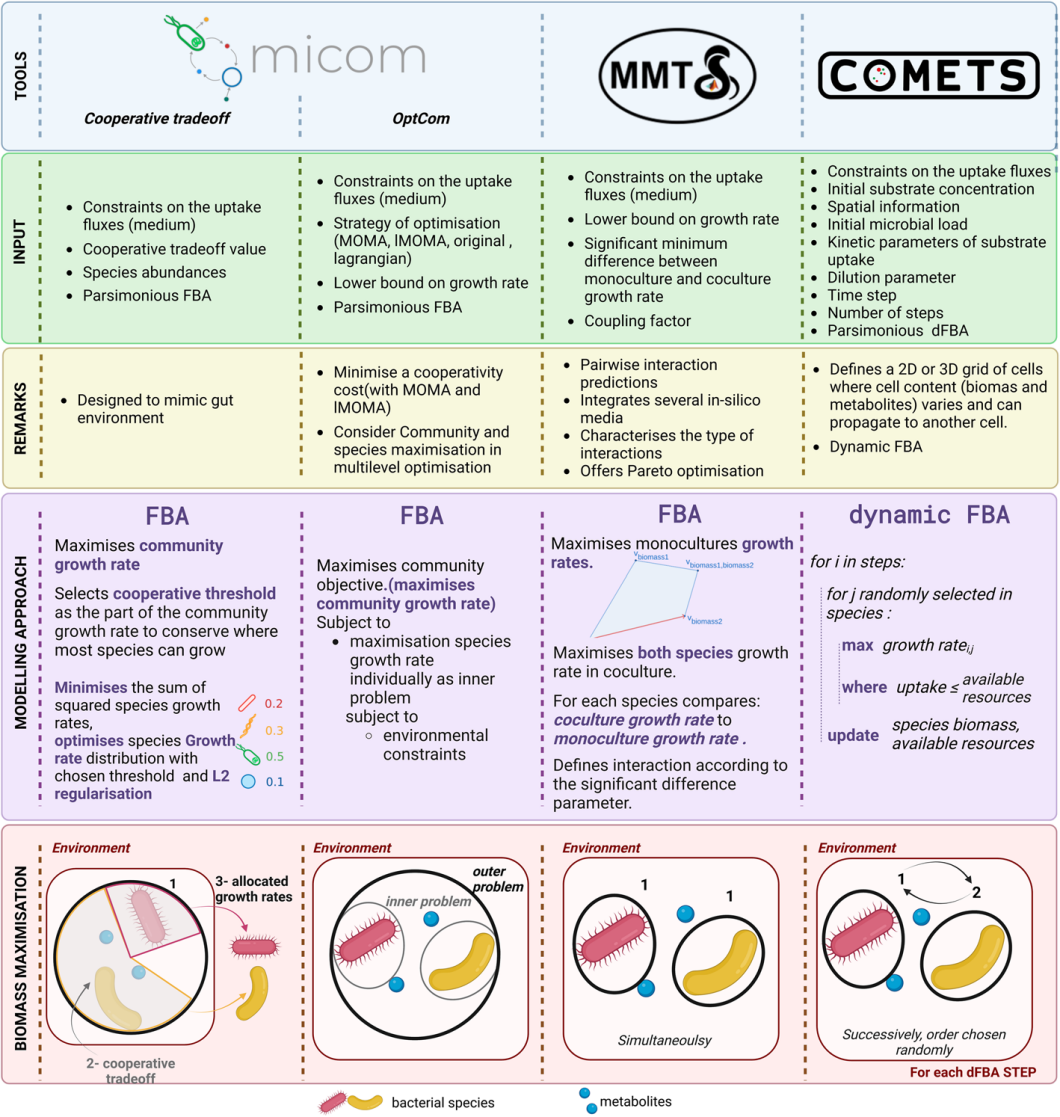
Overview of the tools used in the evaluation. Three tools were selected for the evaluation: MICOM with the cooperative tradeoff function and different OptCom versions, MMT simulatePairwiseinteractions function, and COMETS. The input parameters are further discussed in Table 3 in Methods. The “biomass maximization” panel represents the objective function maximization for all methods. For the OptCom implementation in MICOM, the original strategy of optimisation is shown

We focus on three tools that cover a range of approaches regarding the treatment of the (community) biomass function(s) (see Table 1 and Fig. 1). The Microbiome Modeling Toolbox (MMT) [3] implements a pairwise screen for microbe–microbe and/or host–microbe metabolic interactions that are inferred by determining metabolic exchanges between them. For this, the biomass functions of the species under study are both included in a merged model. Using the merged model, one species is silenced while the growth rate for the other is optimized, and vice-versa (monocultures). Then, a third optimization is performed, maximizing both growth rates simultaneously (co-culture). Given the predicted growth rates in mono- and co-culture, an interaction is reported if the ratio of growth rates in mono- and co-culture is above or below a user-defined threshold. MMT also offers another approach (not evaluated here), where it incorporates relative abundances from sequencing data into the community model. In this case, the community model consists of three compartments (diet—lumen—fecal), and a community biomass reaction is built based on the biomass functions of each of several species present in a sample and on their corresponding relative abundances. In both approaches, the protein-demand reactions, i.e., reactions that enable the accumulation of enzymes, are coupled with their corresponding protein recycling/utilization reactions. This way, the dependency between protein synthesis and utilization is ensured [40].

**Table 1:** Overview of the interaction prediction tools and their parameters

Tool	Input	Community approach	Optimisation aproach	Community biomass function	Output	Special capabilities	Method ID	Tool parameters
COMETS	List of GEMs along with their corresponding initial biomass for each community	Dynamic	Maximizes each species biomass sequentially considering their initial biomass and total uptake then updates species' biomass and extracellular compound concentrations	No	Growth curves = > Growth rates and maximal biomass	Spatial dimension Time dimension Chemostat or batch	H	param = test_tube timeStep = 1 maxCycles = 10 initial_pop = 1 g/sp objective_style = MAXIMISE_OBJECTIVE_FLUX and MAX_OBJECTIVE_MIN_TOTAL
							H/10	param = test_tube timestep = 0.1 maxCycles = 20 initial_pop = 0.002 g/sp objective_style = MAXIMISE_OBJECTIVE_FLUX and MAX_OBJECTIVE_MIN_TOTAL
MICOM	List of GEMs from each community (samples)	Cooperative trade-off	Maximizes community growth rate (sum of the individual growth rates) then, limits community growth to only a fraction of its maximum rate to create a trade-off between optimal community growth and individual growth rate maximization	Yes	Growth rates	Like OptCom but in a more efficient, simpler implementation. Growth rate estimates dependent on species abundance in the community under study	Tradeoff	Fraction = from tradeoff() function pfba = True min_growth = 0 solver = gurobi
		OptCom	Maximizes community growth rate (as described above) by maximizing each species growth at a steady state using a series of strategies (lagrangian, minimization of metabolic adjustment (MOMA), linear MOMA (LMOMA))	Yes	Growth rates	Does not assume that all taxa have the same growth rate; allows for taxon-specific dilution rates	lMoma, Moma, Original	Strategy = (moma, lmoma, original) min_growth = 0 solver = gurobi
MMT	List of GEMs handled in pairwise combinations	Pairwise	Maximizes biomass functions of both species simultaneously using a merged model of the 2 GEMs	Yes	Growth rates, interaction type	User-provided threshold defining what is considered a significant difference between growth rate in mono- and co-culture		sigD = 0.1 c = 400 u = 0 solver = gurobi

Like the community modeling module of MMT, MICOM uses relative abundances derived from amplicon or metagenomic sequencing [19] as a proxy for dry-weight taxon abundances. MICOM can be considered as an extension of the multiobjective OptCom [21], and SteadyCom [20] approaches that simultaneously maximize both the individual and the community growth rates. OptCom acknowledged the existence of a trade-off between growth at the level of the community and its members and addressed it by solving a bilevel optimization problem (one optimization problem embedded within another) where the inner problem pertains to the individual species' biomass production and the outer to the community and its corresponding objective function. Using a computationally simpler approach, MICOM assumes a (constant) growth rate μ_i_ for each species and constrains the overall community growth rate μ_C_, which is obtained by a weighted sum of the individual species growth rates using a trade-off parameter. As there are infinitely many combinations of weights even for a single value of μ_C_, MICOM implements a regularization by implementing an additional function over the individual growth rates μ_i_, requiring consistency with the observed abundances. As shown by the authors, quadratic regularization (L2) fulfills these requirements. Thus, the ‘cooperative trade-off’ approach in MICOM incorporates a trade-off between optimal community growth (maximizing μ_C_) and individual growth rate maximization (L2 minimization). MICOM also supports OptCom-based approaches. More specifically, one can maximize the total community biomass subject to the maximization of every species’ biomass (“original” strategy). Alternatively, one can minimize the cooperative cost, meaning the relative loss of a species to benefit the community, subject to the maximization of the community’s total biomass (minimization of metabolic adjustment; “moma” strategy [41]); the cooperative cost, in this case, is based on the sum of the subtraction of each species' growth rate from its optimal growth. Likewise, the “lmoma” strategy minimizes the cooperative cost considering its absolute value.

The COMETS package [23, 42] introduces two dimensions not considered by MMT and MICOM, namely the physical space (in two or three dimensions) and time. In COMETS, each species has an initial biomass that can optionally be placed in certain location(s). As the environment of a community changes over time due to the consumption or secretion of compounds by the species present, COMETS simulates these changes over time through dynamic FBA [43]. At each iteration, COMETS estimates each species' uptake bounds based on the concentration of nutrients in the medium to check that there is a sufficient amount considering the total uptake rate and optimizes each model using standard FBA. The resulting fluxes are then provided as inputs to estimate the changes in the biomass of each species as well as the concentration of the extracellular metabolites for the next iteration. Therefore, contrary to the MMT module for community modeling and the MICOM approaches, COMETS does not assume a community biomass function.

Here, we evaluated the performance of MMT, MICOM and COMETS on 100 experimentally quantified ecological interactions using 29 metabolic reconstructions (including four high-quality ones) of 25 mammalian gut bacteria (see Fig. 1). Table 1 provides details on the three tools.

## Results

**Fig. 2:**
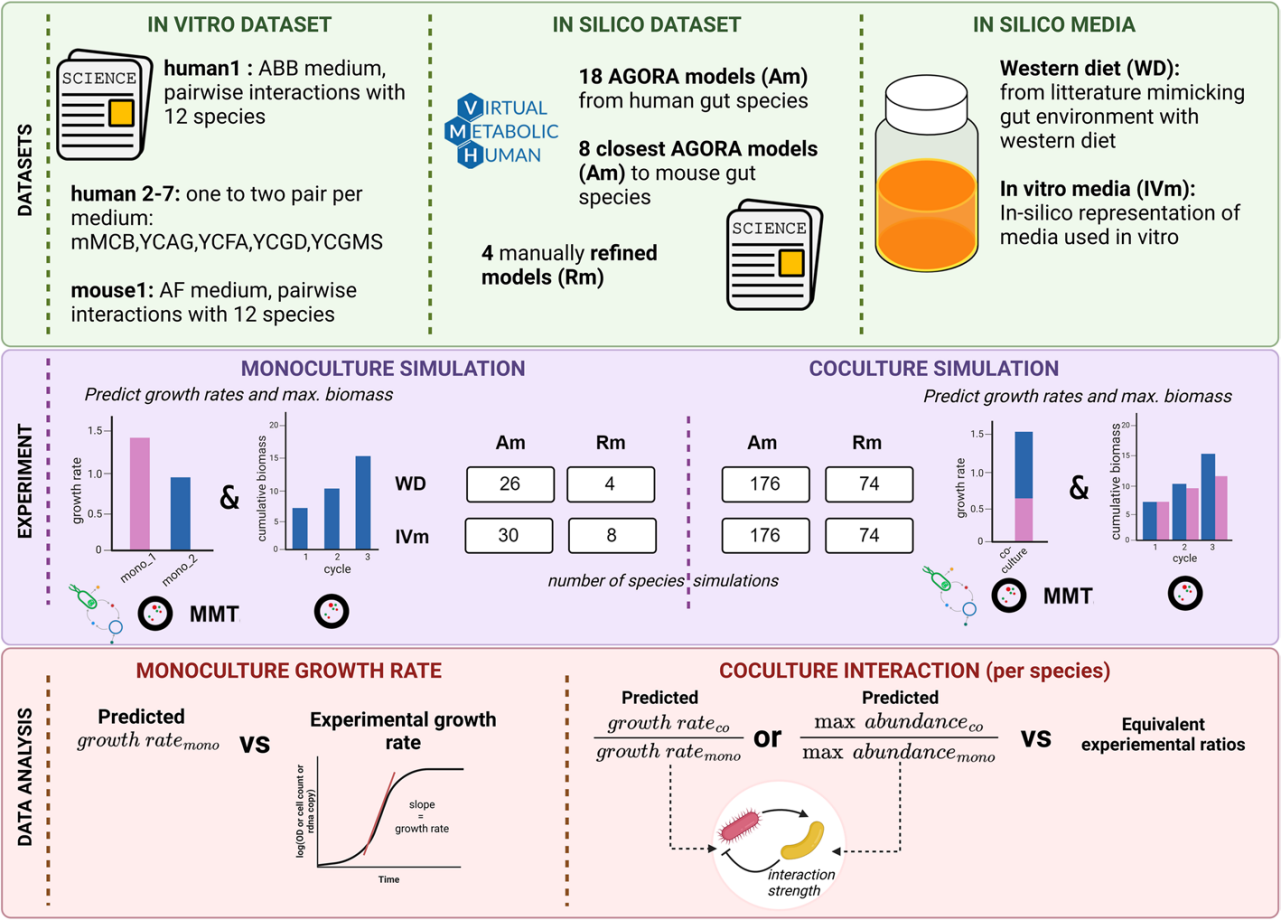
Overview of the process in this analysis: The three software tools (MICOM, MMT and COMETS) were validated using 18 semi-refined models from AGORA (Am) and four refined (i.e. manually curated) models (Rm). Predictions were carried out using in vitro media matching the composition of the media in the experiments with the uptake flux bound values (mmol/gDW/h). The predicted monoculture growth rates were compared to growth rates derived from experimental growth curves as the slope of the exponential phase in log scale. Finally, ratios of growth rates or maximal abundance (the latter only in COMETS) in co- and monoculture were compared to corresponding experimental data in co- and monoculture to evaluate the accuracy of interaction strength prediction

This study evaluated the performance of three different tools (MICOM, MMT and COMETS) with different parameters, two environments including in vitro media (simulations of the ones used in the actual in vitro experiments) and Western diet, and two sets of models (GEMs) with different levels of curation (AGORA models and refined models). First, growth rates were calculated for each monoculture in each condition and compared to the experimental growth rate of the corresponding species to determine a correlation (Fig. 2A, Additional file 1: Table S1 and Additional file 2: Text). Then, co-culture growth rates were predicted using 13 different tool settings across the four conditions (two environments and two curation levels). Interaction strength was quantified as the ratio between either the growth rates or the maximal abundance in co-culture versus monoculture. Predicted ratios were then compared to the experimental ratios of the species growth rate or abundance in co-culture versus monoculture (Fig. 2B, Additional file 1: Tables S2–S3 and Additional file 2: Figs. S1–S2). Here, we present the results related to the AGORA reconstructions using the in-silico representations of the corresponding media used in vitro, as this is common practice [35, 44]. Further analyses using (1) the AGORA models with the Western diet medium, (2) the refined models with the in vitro media simulations and (3) the refined models with the Western diet are available in the Additional file 2: Figs. S1–S2, Additional file 1: Tables S3–S4).

### Monoculture growth rates

**Fig. 3:**
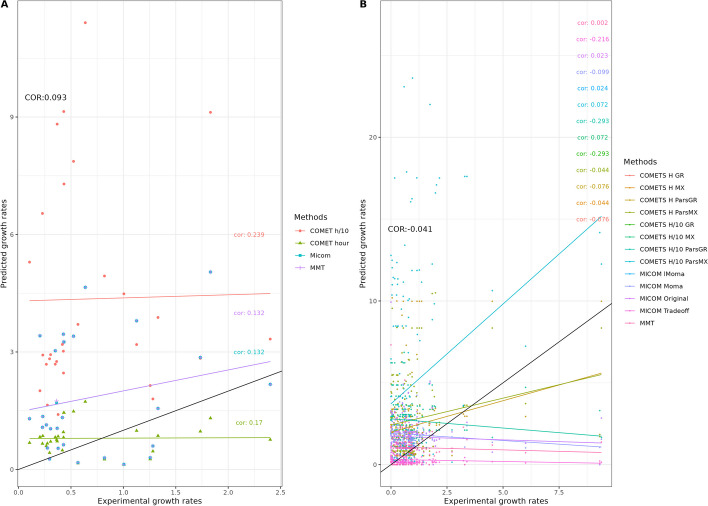
Experimental versus predicted growth rates in simulations matching the media used in the experiments for: **A** mono-cultures and **B** co-cultures with pairs of AGORA reconstructions using several parameters sets for the 3 software tools (see Table 3). The black line indicates where perfect matches between predictions and observations would be positioned. Spearman correlation coefficient values between expected and predicted growth rates are shown in the respective color per method; black coloring denotes the global Spearman correlation coefficient

When the AGORA reconstructions were used along with in silico media that matched the experimental media, there was no significant correlation between the predicted and the expected growth rate (Fig. 3, Additional file 1: Tables S1 and S2), whereas refined GEMs grown in silico on a Western diet resulted in a significant Spearman correlation (Additional file 1: Table S1, see also Additional file 2: Text), which was however driven by an outlier.

We also evaluated the effect of different methods (COMETS, MICOM, MMT) on prediction accuracy in the two environmental conditions. For AGORA models, the agreement between observed and predicted growth rates is low for all tools.

To evaluate prediction accuracy in a species-specific manner, we calculated the mean absolute difference between predicted and observed growth rates for each species across all methods (Additional file 1: Table S2). As expected, our results show that the mean absolute difference per species is lower in simulations matching in vitro media than in the Western diet (in 72% of the species). Interestingly, for a few AGORA models, the difference between predicted and observed growth rates was an order of magnitude smaller than for others, such as *Desulfovibrio piger* (0.455) and *Prevotella copri* (0.576), compared to *Bacteroides thetaiotaomicron* (3.282) or *Clostridium inoculum* (3.558). Furthermore, only the refined models of *Bacteroides thetaiotaomicron* and *Faecalibacterium prausnitzii* performed better than their AGORA equivalents for both conditions (Western diet and in vitro media).

The perfect agreement between MICOM and MMT was expected for monocultures as both carry out standard static FBA for single species. In case of co-cultures, where the approach for the community model varies (Table 1), differing growth rates for each species, and thus interactions, were predicted from each tool.

### Interaction strengths

Next, we assessed how well metabolic modelling tools predict interaction strength. For this, we quantified interaction strength either as the ratio of the growth rate in co- versus mono-culture (MMT, MICOM, COMETS) or as the ratio of the maximal abundance in co- versus mono-culture (COMETS). A positive effect of an interaction partner on a species results in a positive interaction strength (ratio above one), while a negative effect gives a negative one (ratio below one). We note that this measure can result in several interaction strengths per species (as many as there are interactions in which it participates).

Additional file 1: Table S3 summarizes the expected versus observed interaction strengths and Fig. 3B shows their corresponding correlations. For the refined GEMs (see also Additional file 2: Text), predicted interaction strengths were significantly negatively correlated to measured ones for most methods. Overall, the effect sizes (correlation values) were too small to draw a conclusion on the impact of curation level or the medium.

The effect of the methods used was also assessed. Overall, when all the models are taken into account, none of the four methods performed well, with correlation values being below 0.3 (Additional file 1: Table S3). Additional file 1: Table S4 shows the correlation between the expected and experimental ratios for each species across all methods. The best correlation was obtained for the refined *Flavonifractor plautii* (Spearman correlation 0.657) and *Lactobacillus reuteri* (Spearman correlation 0.613) models when using the Western diet; surprisingly, these models had a correlation close to 0 when the matching media for those two species were used. In addition, there were fewer negative correlations than in the per method analysis (a mean of 22.8% negative correlations per species versus a mean of 43.3% negative correlations per method).

ROC curves were generated per method and per species (see Additional file 2: Figs. S3 and S4 in Additional file 2: Text) for three classes representing negative, absent, or positive interactions. The only category that reached an AUC higher than 70% for any class was the refined models when simulated with matching media (Additional file 1: Fig. 3) and merely for the negative reactions. ROC curves per species (Additional file 2: Fig. S4) were only computed for species for which at least two types of interactions were observed. Out of 20 AGORA GEMs, only 2 performed better than expected by chance, whereas 7 refined models (on different media) out of 19 performed better than chance. *Bacteroides ovatus* and *Bacteroides vulgatus* reached the best AUC for positive interactions when simulated with the Western diet (76% and 71%). Concerning the prediction of positive interactions, the refined model of *Faecalibacterium prausnitzii* in Western diet performs the best among all four refined models in all media. In summary, although predicted interaction strengths were not correlated to observed strengths, the ROC curves illustrate that interaction signs are often predicted correctly with curated models.

### Adjustment of medium definition and investigation of metabolite production

So far, we defined the medium through lower bounds for uptake fluxes, which define maximum uptake rates. However, cells often consume nutrients in a non-linear manner, following Michaelis–Menten kinetics. The parameters of this kinetics can be derived from metabolite concentrations, which are often not available. However, one can also improve model performance by fine-tuning lower bounds such that observed growth rates are reproduced. To explore to what extent such finetuning can improve the prediction accuracy, we selected *Faecalibacterium prausnitzii*, for which several growth experiments are available for comparison [30, 32, 36, 45]. We ran the monoculture simulation with the optimizeCBmodel of the COBRA Toolbox [45] in Matlab for this analysis.

We made modifications to the in silico media (ABB, mMCB, YCFA, and YCGD) by reducing amino acid uptake rates, preventing CO_2_ uptake, adjusting sugar uptake rates to match the Western diet definition (“less sugar”), and aligning sugar uptake rates with the YCFAG medium as defined by Heinken et al. [36] in a separate simulation (referred to as “corrected sugar”). The case with less sugar fixed the maximum uptake rate for all carbon sources present in the model to a flux value of ca. 0.149 mmol/(gDW*h) whereas the case with corrected sugar modified the sugar fluxes between 0.2 (for starch) and 18.5 mmol/(gDW*h) (for fructose) depending on the ratio of the carbon source and its molar mass. A metabolite was counted as 'produced' if it had a positive flux in at least 1 FBA solution.

**Fig. 4:**
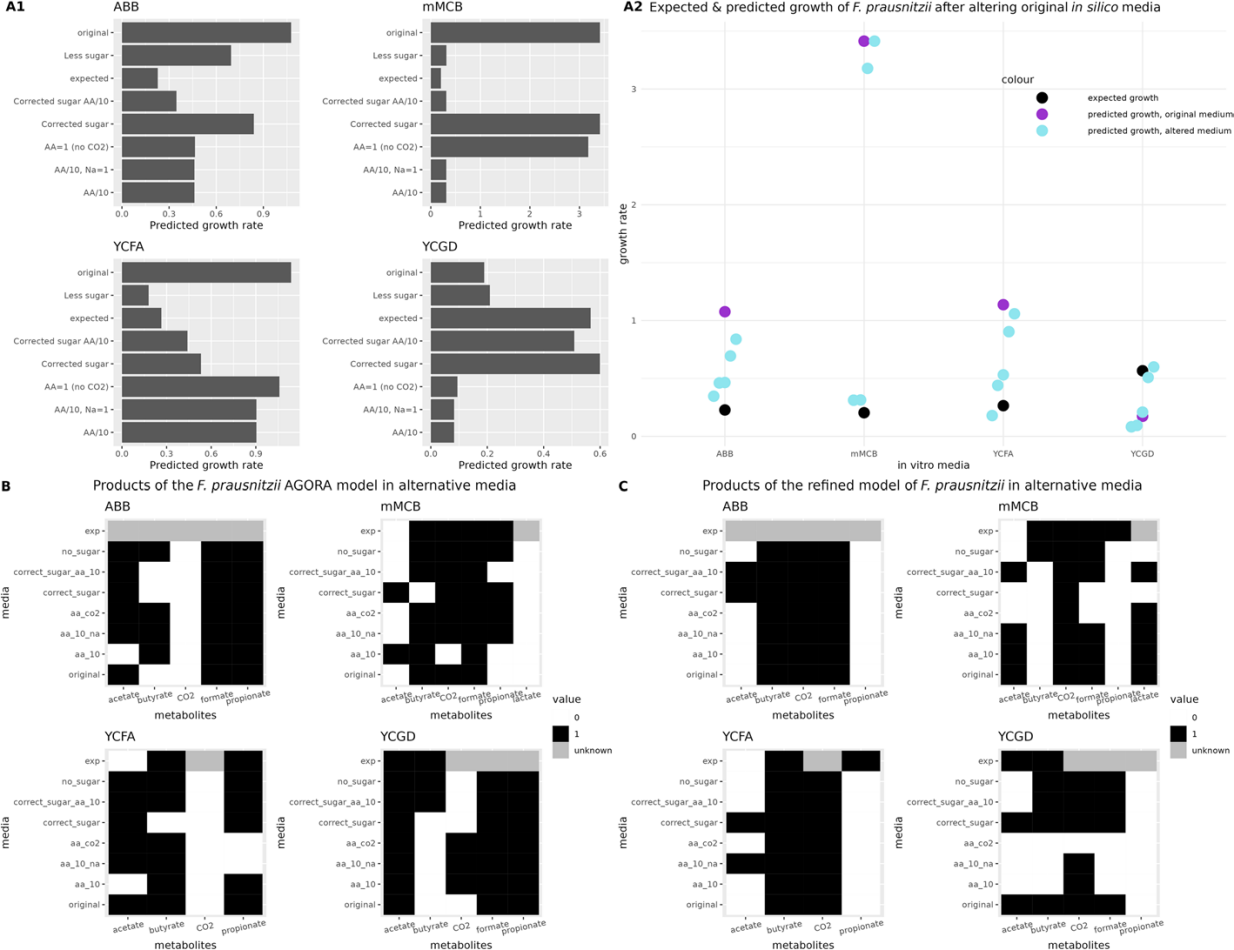
Growth and products of the *Faecalibacterium prausnitzii* AGORA model using different in silico media simulating the ones used in in vitro experiments with modifications. **A1** Growth rates of the AGORA model of *F. prausnitzii* in the in vitro media and their modifications as well as its expected value in each of those. **A2** Comparison of the predicted growth rates from the modifications (cyan) compared to those from the original media (purple) against the expected values (black). **B** Metabolite production (presence: black, absence: white, unknown: grey) of the AGORA *F. prausnitzii* in different media and in their modifications compared to experimental observations. **C** As B but using the refined model of *F. prausnitzii*

The results in Fig. 4 (see also Additional file 1: Table S6) showed that the overestimation of the growth rate, observed in both the refined and AGORA models, was primarily due to the high maximal uptake rates for sugars that were originally set to their mmol/L concentrations assuming unit values for biomass and time. In the ABB and YCGD media, amino acid concentrations were the main factor affecting the growth rate. To address the underestimation of growth rates in the YCGD medium, we increased the maximum uptake rate for sugar to achieve a similar growth rate observed in other media. The mean squared errors of the closest predicted values to the expected ones, from the prediction set of the altered media, equals 0.009 while the one of the initial predictions is 2.984.

Figure 4C and D (based on Additional file 1: Table S6) compare predicted metabolite production to the experimentally observed metabolites. Overall, both the AGORA and the refined model did not significantly differ in how accurately they reproduced known metabolite production. The AGORA models were able to produce propionate and the refined models CO_2_ in most cases. However, the media alterations increased prediction accuracy compared to the original ones; e.g. in the AGORA model case, in the mMCB medium, the predictions using the original in silico medium did not include propionate production, which was the case for several modified media. In summary, the key metabolites were mostly produced as expected, with some variations between the models.

## Discussion

Here, we present the first systematic evaluation of FBA-based interaction prediction for gut bacteria. The evaluation was subject to several limitations. For instance, growth curves were collected with different techniques, such as optical densities and 16S rRNA copy numbers determined by qPCR, which are not free of biases. Optical density is known to saturate at high cell densities, whereas DNA extraction and amplification required for qPCR and 16S rRNA sequencing can differ in efficiency across species [46]. Furthermore, there are a few known cases of bistability, where either one or the other gut bacterium ends up as the sole survivor of the pair, depending on small differences in initial conditions [2]. In addition, there are different methods to calculate growth rates from growth curves, which we did not evaluate here but which may impact results [47].

Defining media accurately is critical, as models require specific metabolites, often vitamins and ions, in varying amounts in the environment. In silico, these metabolites can become limiting factors, even though in vitro bacterial species require only low amounts of vitamins. Furthermore, the number of refined models was too small to reach a strong conclusion on the impact of curation. In addition, we matched mouse gut bacteria to AGORA models of closely related human gut bacteria to work with the same level of curation. However, omitting the AGORA models matched to mouse gut bacteria did not improve the interaction strength predictions (data not shown). Finally, our evaluation was limited to the prediction of growth rates in mono- and bi-cultures and of interaction strengths based on these (except for COMETS, where we also considered maximal abundances). We did not evaluate whether interaction mechanisms were correctly predicted since such mechanisms are rarely systematically explored and mostly unknown.

Prediction accuracy was generally low and, as expected, was not improved by keeping the number of non-zero fluxes at a minimum (pFBA). The minimal cooperative value for the MICOM tradeoff function resulted in low growth rates for both species most of the time. However, it did not systematically perform worse than other MICOM methods. In contrast, shortening the time step in COMETS increased the predicted growth rate, which in some cases positively impacted the interaction strength prediction. As mentioned, each of the tools implements a different approach to community FBA. However, our evaluation did not indicate that one approach systematically outperformed the others. While there was no clear winner, the tools do have unique strengths. For instance, only COMETS implements dynamic FBA and thus was the only tool that could assess interaction strength as the ratio of maximal abundances in co- and monoculture. Interestingly, the maximal abundance ratio outperformed the growth rate ratio when predicting positive interactions with curated GEMs (in IVm, AUC = 0.63). Except for this case, only the prediction of negative interactions performed significantly better than random, with a large variation across species. We observed that the use of curated GEMs significantly increased the accuracy of growth rate prediction. However, this observation is based on only four curated models and driven by an outlier (*Enterococcus faecalis*).

There are several reasons for the low prediction accuracy. The quality of the metabolic reconstruction is an important factor since missing or wrongly assigned reactions can lead to false flux distributions and, consequently, misleading growth rate predictions.

Recently, Schäfer et al. [48] were able to obtain accurate predictions using GEMs after gap-filling draft reconstructions several times using in silico media simulating known growth-supporting carbon sources (45 carbon sources in total). They integrated several alternative reaction sets for each carbon source separately into the draft reconstruction and tested whether they could predict growth in a minimal medium. Notably, combinations of reaction sets from up to 3 carbon sources returned the most accurate model. This highlights the extent to which phenotypic data benefit metabolic modeling.

In addition, the biomass reaction should ideally be based on measurements of macromolecule percentages in the cell but is often transferred from a model organism with only minimal curation [49]. Even if macromolecule percentages are available, these are usually limited to monocultures and may change in co-culture. Interaction prediction with FBA-based methods also has several inherent limitations. Importantly, only metabolite-mediated interactions can be predicted, which means that other interactions, for instance, through the Type VI secretion system or quorum sensing, are outside the scope of FBA. Also, it is unclear how to define interaction strength. In FBA, it is usually computed as the ratio of co- and mono-culture growth rate, assuming cells are in exponential phase [4, 50]. In other phases of the growth curve, the growth rate cannot be quantified easily with FBA. Abundance ratios can be used as an alternative measure, which, however, requires dynamic FBA. Further, extracting growth rates from the in vitro experiments was not straightforward as growth curve data are often not provided in numerical form.

Our conclusions are in line with the findings of Jansma and El Aidy [50], who highlight the need to use condition-specific GEMs. In agreement with this, Magnusdottir et al. [51] compared the relationship between a standard and condition-specific GEM to the one between the genome of a species and its transcriptome. In another FBA tool evaluation, Scott Jr et al. [52] also highlighted the need for tool improvement regarding quantitative predictions.

Future evaluations of FBA-based microbial interaction prediction could include additional tools and approaches not tested here, such as Pareto optimality [53, 54] as well as topological approaches that ignore reaction stoichiometries [26, 55, 56], and FBA approaches integrating further biologically driven constraints (e.g., resource allocation [57], enzyme abundances [58] or Gibbs energy [59]). In addition, it will be interesting to increase the species number per experiment to test methods developed for communities (such as mgPipe in MMT) and to use more curated models to investigate whether curation improves prediction accuracy. Finally, since this evaluation was limited to mammalian gut bacteria, microorganisms from other environments need to be considered.

## Conclusions

We tested several FBA-based interaction prediction approaches on in vitro data of mammalian gut bacteria collected from the literature and found that prediction accuracy is low without further curation or parameter tuning. This finding is a warning that interaction prediction with metabolic models requires model refinement and thus cannot be carried out in a high-throughput manner.

## Methods

### Data

We searched the literature for gut microbiota datasets containing growth curves for mono- and co-cultures. We identified six articles that provided relevant data for our study, which we organized into seven data sets (see Table 2).

**Table 2:** Overview of experimental data

Reference	Data sets	NB species	Medium	Culture condition	For monoculture growth rate		For coculture		
							Monoculture	Coculture	Ratio
Venturelli et al. [2]	Human 1	12	ABB	Serial dilution	*Provided through Lotka Volterra model parameterization*	Calculated from OD (visually extracted)	OD (visually extracted)	OD multiplied with relative abundances from 16S rRNA gene sequencing	Calculated from monoculture OD and coculture OD-multiplied 16S data
									*Interaction coefficient and sign from generalised Lotka Volterra model*
Wang et al. [34]	Human 2	2	YCAG	Batch	*log 16S rRNA gene copy number per ml culture*	Calculated from OD	ddPCR (log 16S rRNA gene copy number per ml, visually extracted)	ddPCR (log 16S rRNA gene copy number per ml, visually extracted)	Calculated from mono and coculture 16S gene copy number
Das et al. [32]	Human 3	2	YCGMS	Batch	Calculated from qPCR (log 16S rRNA gene copy number per ml culture, visually extracted)		log 16S rRNA gene copy number per ml (visually extracted)	log 16S rRNA gene copy number per ml (visually extracted)	Calculated from mono and coculture 16S gene copy number
Cheng et al. [30]	Human 4	2	YCFA	Batch	Calculated from OD (visually extracted)		OD (visually extracted)	*OD*	*No ratio possible*
D'hoe et al. [31]	Human5-6	2_2	mMCB	Batch	Calculated from qPCR (TaqMan)		qPCR (TaqMan)	qPCR (TaqMan)	Caclulated from mono and coculture TaqMan qPCR data
Das et al. [32]	Human 7	2	YCGD	Batch	Calculated from qPCR (log 16S rRNA gene copy number per ml culture, visually extracted)		log 16S rRNA gene copy number per ml (visually extracted)	log 16S rRNA gene copy number per ml (visually extracted)	Calculated from mono and coculture 16S gene copy number
Weiss et al. [33]	Mouse1	8	AF	Batch	Provided (OD)		qPCR (16S)	qPCR (16S)	Calculated from mono and coculture 16S gene copy number

Data not used in the analysis is marked in italics. Data sets five and six share one species

#### Monocultures

When a growth rate was not provided, we calculated it as the slope of the linear part of the growth curve in logarithmic scale, computed between start time point (t_1_) and end time point (t_2_). The abundances are measured as optical density, log 16S rDNA copy number per ml or cell count per ml, and t_1_ and t_2_ are time points chosen in the exponential phase. These were not identical across the curves since the onset of the exponential phase varied across species. In the Human1 data set, the *Eggerthella lenta* growth rate could not be determined because it was too close to the x-axis. The growth rates obtained for each monoculture in vitro are gathered in Additional file 1: Table S7.

#### Co-cultures

To quantify interaction strength, we calculated the ratio of co-culture to monoculture abundance:

$$ratio = \frac{{x_{co} }}{{x_{mono} }}$$

where $x$ represents the maximal abundance in monoculture (mono) or co-culture (co). In the Human1 data set, co-cultures were grown in serial dilution at 24 and 48 h. The authors provided up to three maximal OD values in the stationary phase and three relative abundance values. The transformed maximal OD was obtained for each species by averaging maximal OD values multiplied by the relative abundance. However, in the Human1 data set, *Prevotella copri* never reached the stationary phase, making it impossible to calculate the ratios for this species. Thus, we ignored all co-cultures involving this species in this series. In the Human4 data set, the OD of the co-cultures and monocultures were available, but relative abundance information was not provided. Therefore, we were unable to calculate the ratio for this series. Nevertheless, we included the monocultures for growth rate prediction. In total, we used 30 monocultures and 88 co-cultures (176 ratios) in our analysis. Additional file 1: Table S8 provides the species name used in the literature and the updated species name after a recent taxonomic revision.

### GEMs

To compare the effect of model refinement on prediction quality, we used both semi-refined and refined metabolic models. An overview of the models used is presented in Additional file 1: Table S8.

#### Semi-refined models

The semi-refined models used in this study were obtained from the AGORA [35, 60] project, which provides metabolic model reconstructions for human gut species and is available on the VMH.life website. However, as none of the available models matched the mouse bacterial species or strains used in our study, we computed the Average Nucleotide Identity (ANI) score [61] between the mouse bacterial strain genomes and the genomes used to reconstruct the AGORA models. ANI scores were calculated with the FastANI tool [62]. The AGORA genome with the highest ANI score was considered as the most closely related species to the mouse one, and its corresponding AGORA model was used for the analysis. In all but one case, the ANI score of the genome used was higher than 95%, indicating that the two genomes belong to the same species (Additional file 1: Tables S9 and S10).

As AGORA2 was recently published [60], we repeated the ANI score calculation using the reconstructions of it (Additional file 1: Table S11). For all the query genomes a different genome was found with a higher ANI score than from the ones included in AGORA. Therefore, a different reconstruction was mapped for each strain under study. We performed the MMT analysis with those models and the interaction predictions differed from those using the AGORA models but were not in statistically significant agreement with the expected ones (Additional file 1: Table S12).

#### Refined models

For the refined models, we identified four GEMs that matched species in the experimental data, but only a few were available for gut bacterial species. Two models were found for the human data set, *Bacteroides thetaiotaomicron* (BT_iAH991) [37] and *Faecalibacterium prausnitzii* (iFprauz) [36], and two for the mouse data set, namely *Akkermansia muciniphila* (Yakk_v2)[39] and *Enterococcus faecalis* (enterococcus_faecalisV583) [38]. These refined models were curated by different scientists, using different names for metabolites and reactions, which made them incompatible with each other. To resolve this issue, we modified the exchange reaction and metabolite names in the refined models to match those in the AGORA models. However, some entities in the *Enterococcus faecalis* model had no equivalent in the AGORA model and were left unchanged. Some fields (i.e. annotations) were added to the structure of some refined GEMs to meet the expectations of MMT, which was developed to be used with AGORA models and is designed to be consistent with their structure.

### Medium definition

Accurately defining the environment is critical for reliable metabolic predictions. To assess the impact of environmental complexity, we selected two sets of media for our analyses. The first set is used in metabolic models of human gut microbiota, but it is not representative of the selected experimental conditions. The second set is designed to mimic the in-vitro selected experimental environment.

#### MMT medium

The MMT tutorial offers four media formulations that mimic the gut metabolic environment [35]. We chose the Western diet-like medium without oxygen as the baseline for our analysis since it is compatible with AGORA models (identical reactions and metabolite names). Furthermore, all models were able to grow on the Western diet-like medium except for the refined model of *Akkermansia muciniphila*.

#### In vitro media

We used a total of seven complex media, namely ABB, AF, mMCB, YCAG, YCGMS, YCFA, and YCGD, which were supplied in anaerobic conditions with nitrogen (5–10%), carbon dioxide (10%), and dihydrogen (5–7%) (Table 3). The exact chemical composition of these media is unknown since they include components such as peptones, amino acids, yeast extract, brain heart infusion, and calf serum. To ensure consistency in the composition of peptones and other amino acid mixtures, we assimilated them to have the same composition per gram of amino acid and salt, as described by Microxpress® for soya peptone. Calf serum and heart infusion were supplemented with vitamins to ensure optimal bacterial growth.

**Table 3:** Overview of the parameters used for each of the methods

Method		ID	Name	Description	Value in experiment
MMT		c	Coupling factor	Define how the reaction fluxes are coupled to the biomass reaction flux and defined as: flux span = − (c * flux(biomass)) to + (c * flux(biomass))	400
		SigD	Significant difference	Value that counts as a significant difference between monoculture and coculture growth rate	0.1
		u	Threshold u	Flux allowed in reactions if biomass flux = 0	0
		mergeGenes	Gene merging variable	Boolean, wether the gene are added and merge in the community mode (Time consuming)	FALSE
MICOM	cooperative_tradeoff	cooperative_tradeoff(= fraction)		Minimum proportion of the maximal community growth to allocate to species growth rate	Min value where both species are growing between 0.1 and 1 with 0.1 step
		pfba	Parsimonious FBA	Define if a parsimonious FBA is performed	TRUE
		min_growth	Minimum growth rate	Minimum growth rate required for each species	0
		Abundance		Relative abundance of each species in the community. By default each species has the same abundance	0.5 and 0.5
	OptCom	Strategy		Strategy used to solve the optimization problem	MOMA
					lMOMA
					Original
		min_growth	Minimal growth	Minimal growth required for each species	0
		pfba	Parsimonious FBA	Define if a parsimonious FBA is performed	TRUE
COMETS	H/10	initial_pop		Gram of biomass in the environment	0.002
		time_step		Time step of a FBA problem in hour	0.1
		maxCycles		Number of steps max	20
	H	initial_pop		Gram of biomass in the environment	1
		time_step		Time step of a FBA problem in hour	1
		maxCycles		Number of steps max	10
	BOTH	defaultVmax		V max value per default in mmol/g. CDW/h	18.5
		defaultKm		KM value per default in M (molar conc.)	0.000015
		SpaceWidth		size of the cell in cm3	1
		maxSpaceBiomass		Capacity maximum in gr. cell dry weight	100
		minspaceBiomass		Capacity minimum in gr. cell dry weight	1.00E−11
		obj_stype	Objective type	Define if the strategy used to solve the optimization problem	MAX_OBJ_MIN_TOTAL = > Pfba AND MAXIMIZE_OBJECTIVE_FLUX = > pFBA
		Grid	Grid size	Number of boxes in the x and y axis to define the grid size	[1,1]
		Static		Related to metabolites in media definition, define if the metabolites are in limited amount	TRUE

Parameters not shown here were kept at their default value in the calculation

For tryptone and yeast extract, we followed the protocol described by Marinos et al. [63] for GEMs and converted the flux constraints according to the initial quantity of each in the respective medium. The concentration of other components was provided either in molar or gram per liter units, which we converted to molar mass. The molar concentration (mol/L) was used as flux value (mmol/gdW*h).

We modified the in vitro media to ensure optimal bacterial growth, using the Western diet as a reference for most bacterial models. The yeast extract and tryptone defined in silico contained oxygen, which was not compatible with the anaerobic conditions of the experiments, and so was removed. Additionally, although selected vitamins and ions were added to the composition of some complex components, such as calf serum and heart infusion (e.g., vitamin B12, thiamin, riboflavin, biotin), it was not enough to ensure the growth of each species in silico. Therefore, vitamins were added at a quantity judged not to be limiting for bacterial growth (1 g/L) in amounts equal to the ones described in the MMT Western diet medium. Furthermore, some GEMs required other specific metabolites to initiate minimal growth, such as arabinose for *Eggerthella lenta*, N-Acetyl-Neuraminic Acid and 5,6-Dimethylbenzimidazole for *Akkermansia muciniphila* and for *Enterococcus faecalis*. These metabolites were also added to the media at a concentration of 1 g/L, following the amount described in the Western diet medium. The complete description of each medium can be found in Additional file 1: Table S13.

### Tools

The scripts for the three methods are available through a GitHub repository. The scripts were executed using MATLAB R2022a or Python 3.10, and all methods were used with Gurobi 9.5.1. Table 3 shows the complete set of parameters values used.

#### Microbiome Modeling Toolbox (MMT)

The “Computation and analysis of microbe-microbe metabolic interactions” tutorial [64] was followed to predict pairwise interactions with MMT. This method takes as input a list of GEMs and their paths, as well as a list of media or dietary conditions containing the exchange reactions, the lower and the upper bounds. Then, MMT performs all pairwise co-cultures and calculates both monoculture and co-culture growth rates.

The minimum growth rate difference between mono and co-culture that is considered significantly different (sigD value) was initially set to 0.1 (10%). This allows identifying the type of interaction between the two species, but this parameter was not used here since only ratios were considered for this analysis. The coupling factor (c) was kept at its default value of 400, and the threshold (u), defining the flux value allowed through reactions if biomass is null, was set to zero.

#### MICOM

We used MICOM [65] to predict community growth rates. We provided the identifiers and filenames of the species in pairs and then created a community and furnished the medium to parameterize the model. The MICOM medium is defined as a list of exchange reaction names of the community associated with the available flux of the metabolite (positive values). The monoculture growth rates were obtained by creating a community with only one species using the function optimize_single. The optcom function was used with the three strategies lMoma, Moma, and original, with minimal growth of 0 and pFBA. The community prediction was run with the Gurobi solver. The cooperative_tradeoff function was used for the community, with a fraction value that was defined using the tradeoff function and taking the smallest tradeoff value that allowed both species to grow, without consideration for the value of the growth rate, to minimize the cooperation between species. The minimal growth rate was also zero for this function and calculated with pFBA. Finally, the growth rates were extracted from the output.

#### COMETS

COMETS implements dFBA and takes as input the metabolic model of the community and the medium, defined as a dictionary of the metabolites in the medium and their corresponding molar concentration. We followed the comestpy "Growth in a test tube" tutorial [66]. COMETS was run with two settings for mono and co-cultures: For the H (hour) condition, an initial biomass of one gram was used with a time step of one hour. This approach was expected to yield comparable outcomes to other techniques that use a biomass function computed per gram of dry weight and hour. As for the H/10 condition, the initial biomass was set to 2 mg with a time step of 0.1 h. COMETS was run for both settings with both FBA (obj_style: MAXIMIZE_OBJECTIVE_FLUX) and pFBA (obj_style: MAX_OBJECTIVE_MIN_TOTAL). In general, the maximum number of cycles was set to 20 but was increased for some monocultures to ensure that the stationary phase was reached. The other community parameters were the same as described in the tutorial.

### Predictions

All three tools predicted monoculture and co-culture growth rates for 30 AGORA monocultures and 89 pairs. Additionally, monoculture predictions were made for four refined models (four for the Western diet and eight for in-vitro media) and co-culture predictions were made for 76 co-cultures using one of the two available refined models. All predictions were made using the Western diet and one of the seven media that matched the experimental conditions. Finally, the ratios were calculated based on the growth rates obtained from these predictions.

### Statistics

For each species, the effect of growth in co-culture was quantified by computing the ratio of its growth rate in co- versus monoculture. When both a curated and a semi-curated GEM were available for a species, both were included in the analysis. Statistical values were computed using R 4.1.3 and Python 3.10.

#### Spearman correlation

To assess correlations based on both values and ranks, Spearman’s correlations were computed for monoculture growth rates and co-culture ratios (predicted versus experimental). For each of the four groups of combinations (Western diet and AGORA models, Western diet and refined models, in-vitro media and AGORA models and in-vitro media and refined models), a global correlation value was calculated without separating the species or the methods, highlighting the impact of different media and levels of refinement of the prediction. The ‘cor.test’ function in R was used to calculate Spearman correlations and their *p* values.

#### Wilcoxon test

The Wilcoxon test was performed with the R function wilco16xon.test. For all conditions, the mean absolute difference was calculated between the predicted and experimental growth rate for monocultures and between the predicted and experimental ratio of growth rates for cocultures. A paired Wilcoxon test was then carried out to compare AGORA and refined models in both media conditions.

#### ROC curves

We used a multiclass ROC curve to handle three interaction sign classes: positive, neutral, and negative interactions. We arbitrarily defined these classes for non-neutral effects, at 20% of the difference between mono- and co-culture. Consequently, ratios under 0.8 were designated negative, ratios over 1.2 were designated positive, and ratios in between were classified as neutral. Using the *sklearn* Python package's roc_curve function, we computed the true positive rate (TPR) and false positive rate (FPR) for each threshold value. The threshold represents the value above which an interaction strength is classified as belonging to the positive class and below which it is classified as belonging to the negative class. The maximum number of thresholds is the number of ratios per method plus one. We employed the same range of threshold values to compute the TPR and FPR for each class within a method.

### Supplementary Information

**Additional file 1: Table S1**. Overview of statistical values for growth rates predicted in monocultures. H: time step of 1 per hour, H/10: time step of 0.1 per hour. **Table S2**. Mean absolute difference per species across the four methods and for the different media. **Table S3**. Global Spearman correlation refers to the correlation across all methods and settings. Significant *p* values are highlighted in grey. GR: Interaction strength computed as the ratio of growth rates, H: time step of 1 per hour, H/10: time step of 0.1 per hour, MX: interaction strength computed as the ratio of maximal abundances, Pars: Parsimonious FBA. **Table S4**. Correlation between predicted and measured interaction strengths for each species, with those that have refined metabolic reconstructions highlighted in grey. Interaction strength is quantified by comparing the growth rate or maximal biomass in co- and monoculture. **Table S5**. Wilcoxon test value for monocultures and cocultures with refined versus AGORA Models. **Table S6**. Products returned from FBA using the AGORA and a refined model of *Faecalibacterium prausnitzii* using in vitro media and alterations of those. **Table S7**. Experimental growth rates for monoculture species and bacterial load in media. Growth rates for the ABB, YCAG, YCFA, YCGD and YCGMS media, were obtained manually. Growth rates are expressed in h^−1^. **Table S8**. Description of the GEM used for each species with its structure description and MEMOTE score with species names from the literature used for experimental data and modelling and their updated names (nomenclature update 12/22). **Table S9**. Mouse strains and their matched AGORA metabolic models. The selection was based on the highest ANI score between each mouse strain genome and the AGORA models’ corresponding genomes. **Table S10**. ANI score for alignment of mice species against AGORA model species (only top 5 ANI scores shown). ANI scores below 80% were not considered in this analysis. **Table S11**. ANI scores of mice species against AGORA2 model species. With green highlight the genomes included in AGORA2 that have the highest ANI score with the genome of interest, with orange the corresponding ones included in AGORA v1. **Table S12**. Comparison of the growth rates returned from MMT using pairs of mice AGORA and AGORA2 models. R1 stands for the growth of species A and R2 for the one of species B. **Table S13.** In silico media description (IVm and WD) where values represent the lower bounds of uptake fluxes. The lower bounds are set as the mmol/L concentration of every metabolite in the experimental medium assuming unit values for biomass and time.

**Additional file 2: Text**. Findings regarding the experiments using the AGORA models along with the Western diet and the refined models with both the in vitro media and the western diet. Supplementary Figures are included.

## Data Availability

Other informations can be found in the GitHub repository: https://github.com/Clem-Jos/pairwise_tool_comp which contains the experimental and predicted ratio and growth rate values, the scripts for MICOM, COMTES and MMT, the ROC curves produces for all species methods for all conditions.

## Abbreviations

Am: AGORA model (semi-curated GEM obtained from AGORA)
AUC: Area under the curve
FBA: Flux balance analysis
GEM: Genome-scale metabolic model
IVm: In-vitro media
MMT: Microbiome Modeling Toolbox
pFBA: Parsimonious FBA
GR: Growth rate
Rm: Refined model (curated GEM)
WD: Western diet
