# Supplementary material for: Predicting microbial interactions with approaches based on flux balance analysis: an evaluation
Source: BMC Bioinformatics. 2024 Jan 23;25:36. doi: 10.1186/s12859-024-05651-7 (PMC10804772; doi:10.1186/s12859-024-05651-7)
Supplement: Supplementary file 2 — Additional file 2: Text. Findings regarding the experiments using the AGORA models along with the Western diet and the refined models with both the in vitro media and the western diet. Supplementary Figures are included. [file 12859_2024_5651_MOESM2_ESM.docx]

## Additional file 2: Text

Simulation predictions of AGORA models with Western Diet and of refined models with both Western and *in silico* representations of the media used in the *in vitro* experiments.

Using the 4 refined models and for the case of monocultures, COMETS with shorter intervals between iterations (1/10) performed best for WD (Figure 1.B) and MMT for *in vitro* media (Figure 1.C). The significantly increased accuracy of growth rate prediction compared to the AGORA models though could be explained from the small number of models tested (4) and/or by an outlier (*Enterococcus faecalis*). Using the refined models, eight growth rates could be obtained since the growth of *Faecalibacterium prausnitzii* was predicted in four media, growth of *Bacteroides thetaiotaomicron* in two and growth of *Enterococcus faecalis* and *Akkermansia muciniphila* in one medium each. The refined GEMs of *Enterococcus faecalis* and *Akkermansia muciniphila* are the only cases where the mean absolute difference per species was lower when using the Western diet compared to the in vitro medium (Supplementary Table 2).


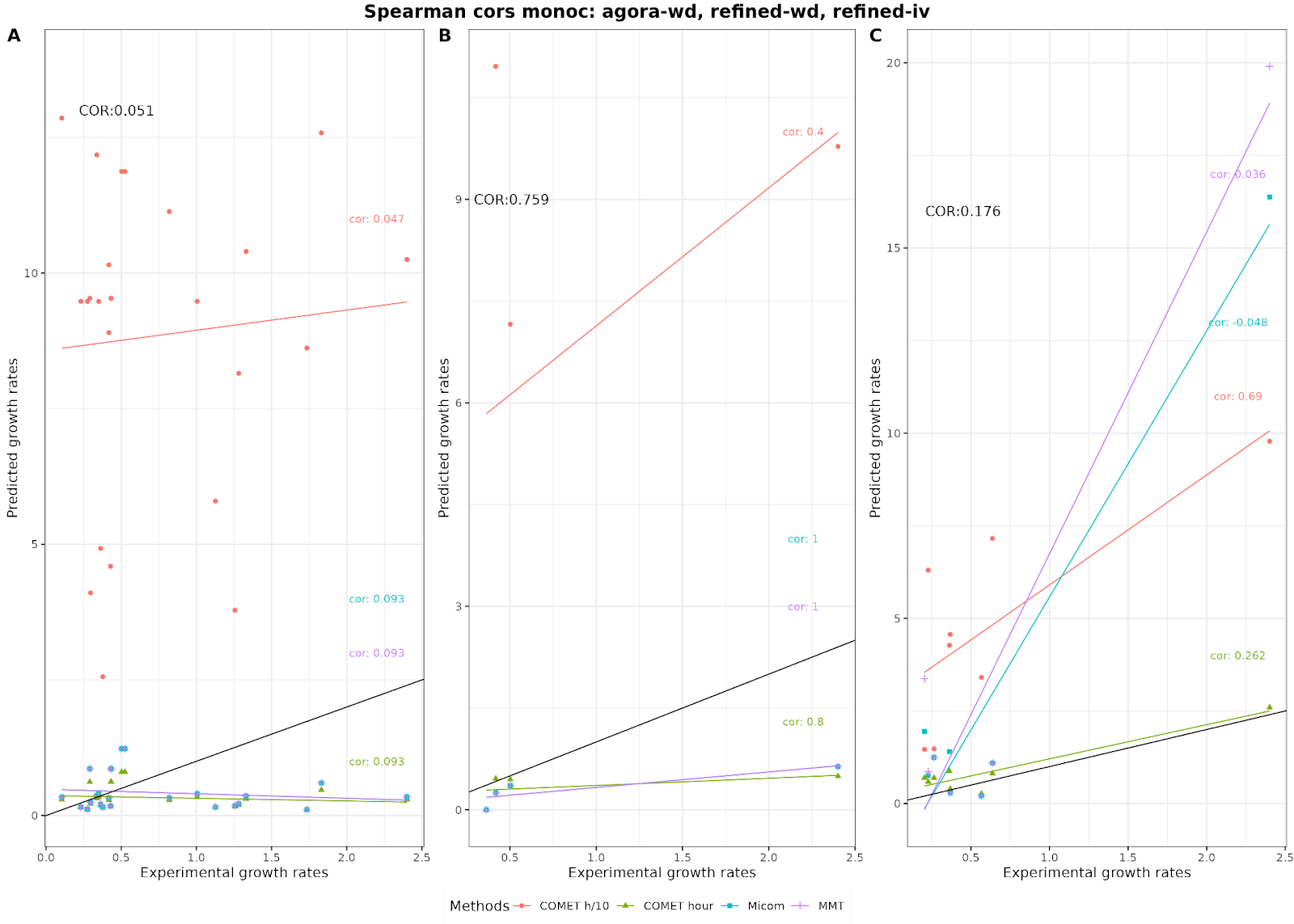


**Supplementary Figure 1:** Predicted growth rates versus experimental growth rates. A: With AGORA models and Western Diet. B: With refined models and Western Diet. C: With refined models and in-vitro media. Colours encode for methods; black is the expected correlation.

With respect to the interaction strength simulations, using the refined models with the simulations of the *in vitro* media, the only significant positive correlation was obtained for COMETS in the same conditions (Figure 2C).

The differences between predicted growth rates (monocultures) and ratios (co-cultures) and corresponding experimental data were also computed and compared for the four conditions (i.e. using the AGORA or the refined models either with the Western diet or with their corresponding *in vitro* media) across all methods with a Wilcoxon test (Supplementary Table 5). While the growth rate difference in the *in vitro* media and the Western dietI was not significant for the monocultures for both the refined and the AGORA models, predicted ratios for the cocultures with the refined models were significantly closer to the expected ratios compared to predicted ratios with AGORA models (Supplementary Table 5). Correlations were also calculated after removing the predictions from mouse GEMs since the GEMs were built for species that did not exactly match the species in the experiment; however, the overall conclusion remained the same.

The refined models, when used with their corresponding *in vitro* medium, gave the best result for MICOM (across all settings, but best result was achieved with Moma, AUC = 0.82), followed closely by MMT (AUC = 0.79). However, those methods were only able to predict negative interactions.


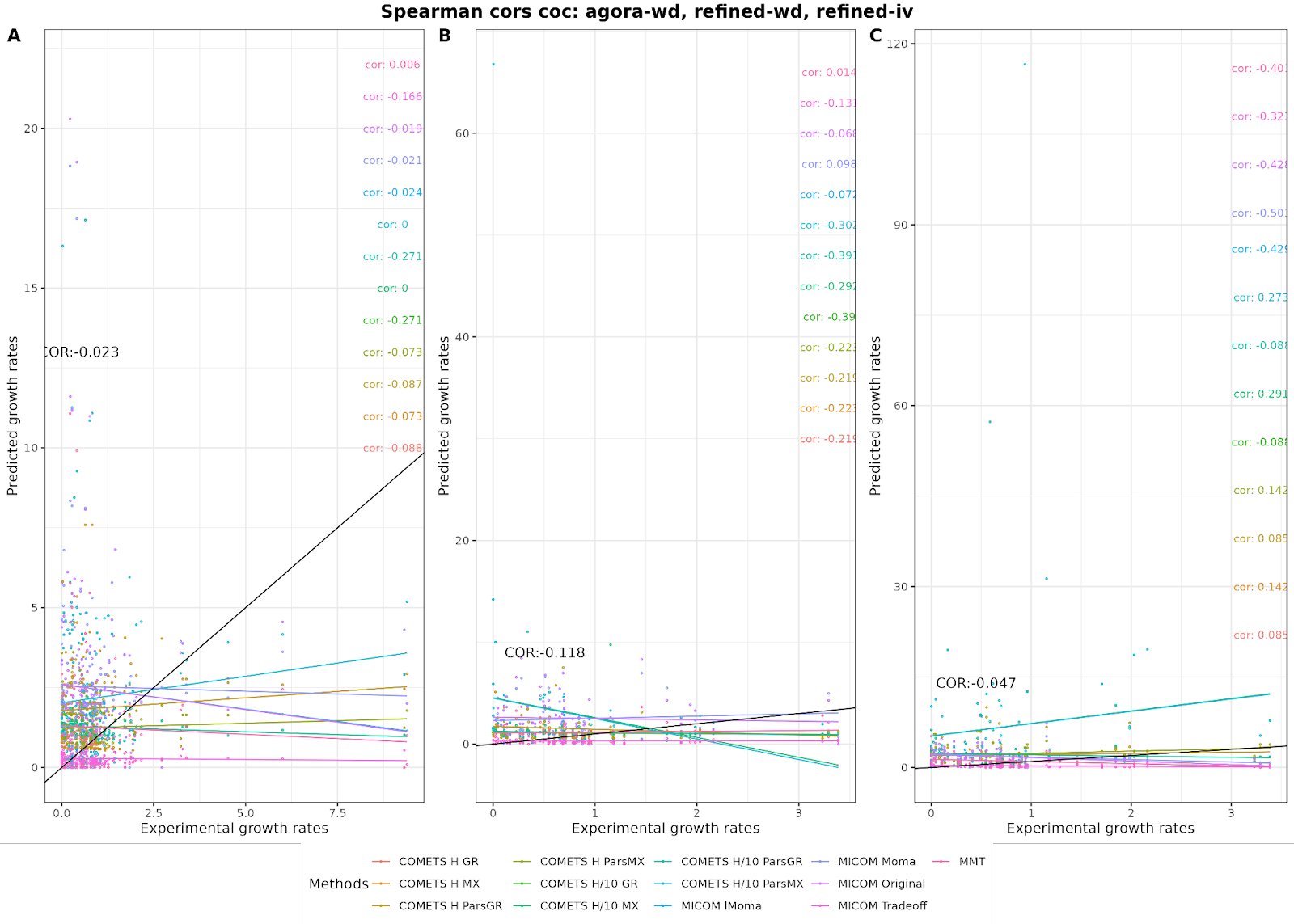


**Supplementary Figure 2:** Predicted growth rates of pairs of species versus experimental growth rates for four conditions. A: With AGORA models and Western Diet. B: With refined models and Western Diet. C: With refined models and in-vitro media. Colours encode for methods; black is the expected correlation.


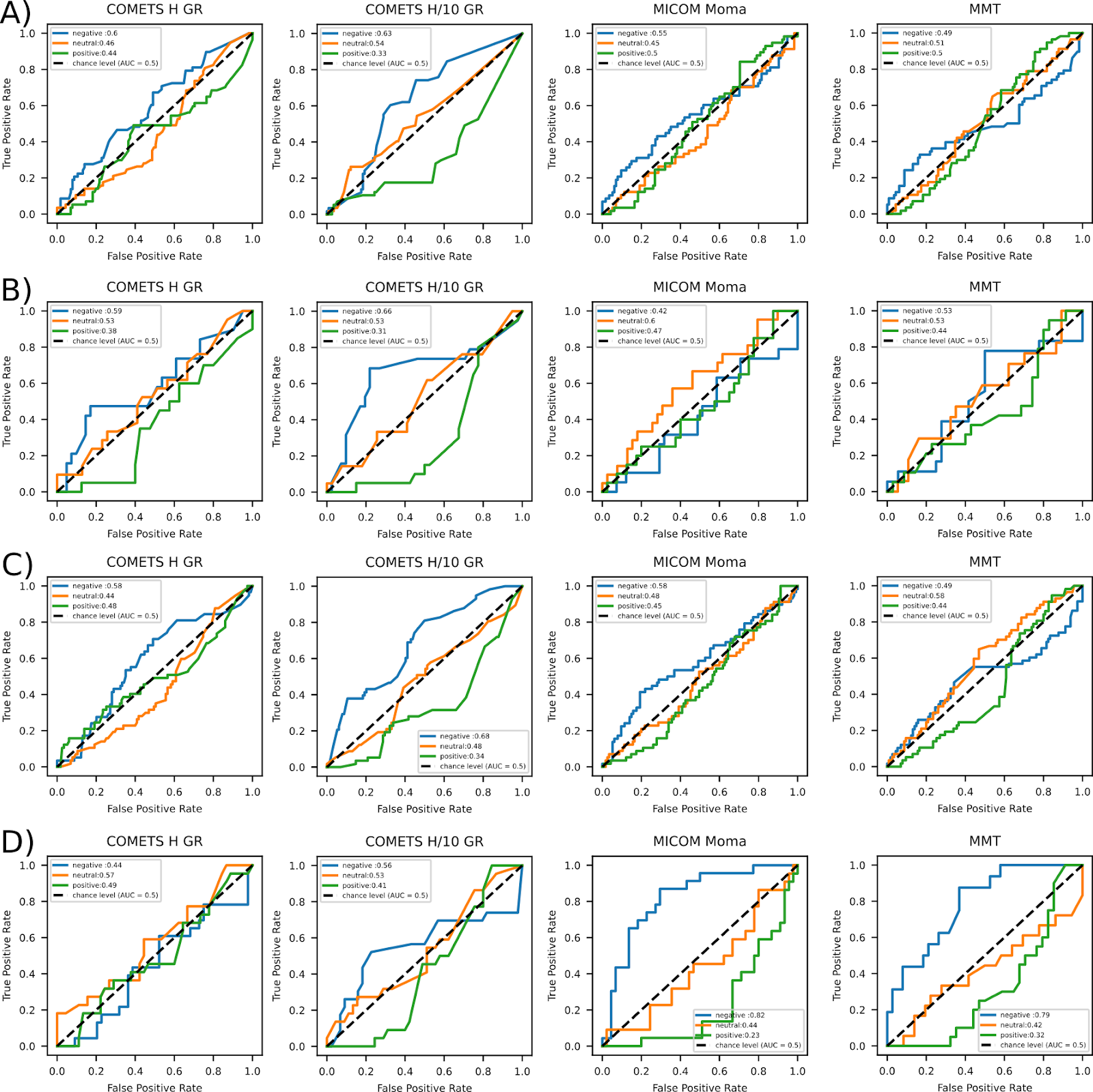


**Supplementary Figure 3:** ROC curves per method (best parameter for each method) with three classes of experimental interactions defined as negative (expected ratio <0.8), neutral (0.8<expected ratio<1.2), and positive (expected ratio>1.2) analysed with a one versus all approach for each of the four conditions tested in this study. A: Western diet and AGORA models, B: Western diet and refined models, C: *In vitro* media and AGORA models, D: *In vitro* media and refined models.


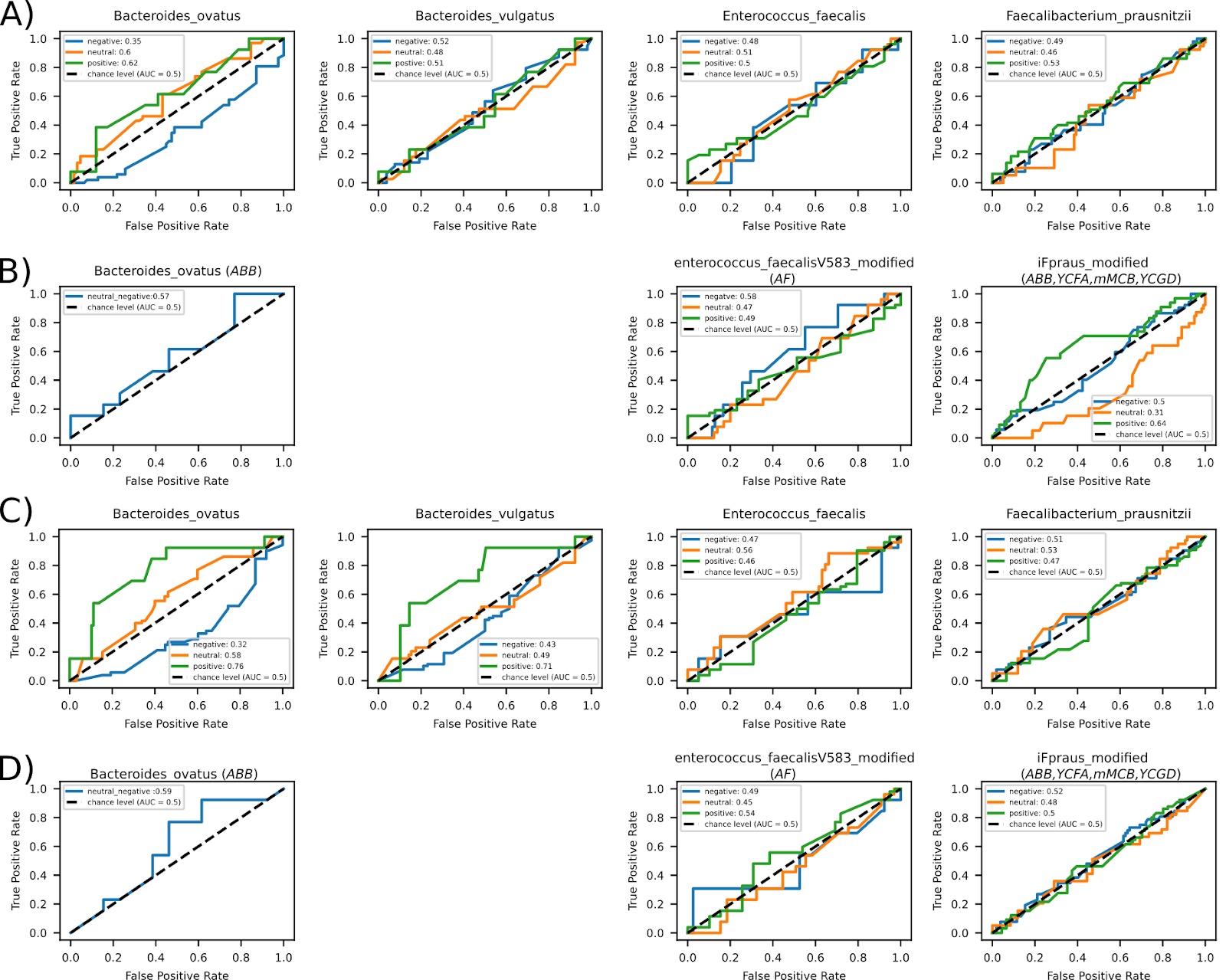


**Supplementary Figure 4:** ROC curves per species (two best and two with refined model) with three classes of experimental interactions defined as negative (expected ratio <0.8), neutral (0.8<expected ratio<1.2), and positive (expected ratio>1.2) analysed with a one versus all approach for each of the four conditions. A: Western diet and AGORA models, B: Western diet and refined models, C: *In vitro* media and AGORA models, D: *In vitro* media and refined models. No curve could be computed for *Bacteroides vulgatus* with a refined model since the expected ratio concerned only belonged to one class. For B and D, media are given between brackets.
